# Prognostic value of baseline and longitudinal changes in exercise capacity and quality of life in the HF-Wii Swedish population

**DOI:** 10.1136/openhrt-2026-004130

**Published:** 2026-07-08

**Authors:** Joanna-Maria Papageorgiou, Tiny Jaarsma, Kristofer Hedman, Nadine Karlsson, Leonie Verheijden Klompstra, Anna Strömberg

**Affiliations:** 1 Department of Health, Medicine and Caring Sciences, Linköping University, Linköping, Sweden; 2 Clinical Department of Cardiology in Linköping, Region Östergötland, Linköping, Sweden; 3 Clinical Department of Clinical Physiology in Linköping, Region Östergötland, Linköping, Sweden

**Keywords:** HEART FAILURE, Outcome Assessment, Health Care, Cardiac Rehabilitation

## Abstract

**Background:**

This study aimed to explore whether baseline values and 12-month change in the 6-Minute Walk Distance (6MWD) and the physical scale of the Minnesota Living with Heart Failure Questionnaire (MLHFQ) were associated with long-term mortality in patients with heart failure (HF).

**Methods:**

A total of 333 patients with HF, enrolled in the HF-Wii trial (NCT01785121) in Sweden, were included. Data on long-term mortality were gathered 3 years after study completion of the last patient included. Study participants were stratified into three groups based on baseline results of the 6MWD: group 1: ≤383 m, group 2: 384–450 m and group 3: ≥451 m. Similarly, tertiles were formed according to baseline MLHFQ physical domain scores: group 1: 0–5 points, group 2: 6–12 points and group 3: 13–40 points.

**Results:**

During a median (25th–75th percentiles) follow-up of 1900 (1566, 2271) days, 48 patients died (14.4%). Patients in group 1 of 6MWD displayed a more severe clinical status in comparison to patients in groups 2 and 3. Kaplan-Meier survival analysis showed a statistically significant between-group difference in survival, observed per tertile groups of the baseline 6MWD. However, no significant difference in survival was found in relation to changes in 6MWD over time. Likewise, there were not statistically significant between-group differences in survival per tertile groups of the baseline score on the physical domain of MLHFQ nor with respect to change over time of the same score.

**Conclusions:**

Baseline 6MWD demonstrated significant prognostic value for long-term mortality in patients with HF whereas the physical score of MLHFQ, as well as their 12-month changes did not reach statistical significance.

**Trial registration number:**

NCT01785121.

**WHAT IS ALREADY KNOWN ON THIS TOPIC**

- Heart failure (HF) is a complex condition requiring a comprehensive approach for accurate patient evaluation with exercise capacity and N-terminal pro-B-type natriuretic peptide established outcome predictors.

**WHAT THIS STUDY ADDS**

- This study confirms that baseline assessment of 6-Minute Walk Distance, in clinically stable patients is a significant predictor of long-term mortality, while changes over time in exercise capacity and Health-Related Quality of Life (HRQoL) in terms of Minnesota Living with Heart Failure Questionnaire (MLHFQ) are not. It adds incremental insight by showing that baseline assessment may be more prognostically informative in comparison to longitudinal changes. Although MLHFQ was not independently predictive, its combined use with functional measures supports a more patient-centred evaluation.

**HOW THIS STUDY MIGHT AFFECT RESEARCH, PRACTICE OR POLICY**

- These findings support combining simple baseline functional assessment with HRQoL measures to enhance clinical evaluation and patient-centred care in HF.

## Introduction

 Heart failure (HF) is a chronic multifactorial clinical syndrome resulting in impaired quality of life (QoL) and high healthcare costs.^1^ It is addressed as a modern epidemic due to an estimated global prevalence of ~2%, with a continuously increasing prevalence due to population ageing. Mortality rates in patients with HF are high, with a 1-year mortality in patients in New York Heart Association (NYHA) class IV up to 50%.^2 3^ The HF trajectory remains unpredictable including intermittent acute and life-threatening deteriorations with gradual decline in functional capacity.^4^ Reliable predictors for long-term outcomes are therefore essential to assist clinicians in tailoring individual therapies and follow-up strategies, as well as motivating patients to achieve higher adherence to treatment. That would facilitate clinicians to act proactively when identifying patients at increased risk of unplanned hospital admissions or Implantable Cardioverter-Defibrillator shocks. Early evaluation and identification of high-risk patients facilitates offering closer monitoring and giving the chance to patients to stay at home as long as possible, considering referral for advanced HF care or even planning for patients’ palliative care. Such proactive measures are supported by European Society of Cardiology (ESC) guidelines advocating early intensified care for high-risk HF patients.^5^ Improved prognostic assessment also enhances shared decision-making and helps align patients’ expectations.^6^

Exercise capacity is one of the strongest predictors of survival in patients with HF.^7^ While cardiopulmonary exercise testing (CPET) is the gold standard for the evaluation of aerobic capacity, the 6-Minute Walk Test (6MWT) is a broadly available, simple and inexpensive test with high reproducibility. It assesses patients’ physical capacity with a high predictive value for mortality in patients with HF,^8 9^ it is considered as an alternative to CPET for risk stratification in patients with HF^10^ and is recommended for risk stratification and follow-up in patients suffering from Pulmonary Arterial Hypertension.^11^

Health-related quality of life (HRQoL) is poor in patients with HF and a low HRQoL is a significant predictor of worse long-term outcomes.^12^ Several studies have shown that clinicians have difficulties in estimating patients’ limitations correctly, resulting in underestimating their needs.^13^ The use of patient-reported outcomes (PRO), such as Minnesota Living with Heart Failure Questionnaire (MLHFQ), has been increasing and has shown a prognostic significance that may help further decision making based on risk stratification.^14^

As mentioned above, HF is a multifactorial condition requiring a comprehensive approach for accurate assessment and management. Single predictors, while valuable, can fail to capture the full spectrum of clinical outcomes. Therefore, the use of multiple predictors is preferable. Combining the 6MWT with HRQoL gives insights into both objective and subjective aspects of HF, allowing a more nuanced and patient-centred approach.

Our study aimed to explore, in a Swedish population with chronic HF, whether there is a prognostic value on long-term mortality of the baseline value in 6MWT and HRQoL, as well as their change from baseline to 12 months.

## Methods

### Study design and patient characteristics

#### The HF-Wii study

The present study is a secondary analysis of data of the HF-Wii study, including only individuals from the Swedish patient population of the main study cohort, in order to be able to linkage with the Swedish Cause of Death registry.

The HF-Wii study was an international multicentre randomised controlled intervention trial performed in six different countries (Sweden, Italy, the Netherlands, Israel, Germany, the USA) from September 2013 to April 2017. The aim of this study was to examine the effects of access to a home-based exergame (Nintendo Wii) in patients with HF on exercise capacity, self-reported physical activity and PRO measures.^15^

In the HF-Wii study, all adult patients with a confirmed HF diagnosis according to ESC (independent of left ventricular ejection fraction), already in contact with a HF clinic were evaluated on inclusion and those found eligible received study information. After the informed consent was gathered, patients were evaluated with the planned tests and completed questionnaires. All tests were made according to a pre-scheduled detailed protocol. Evaluations were repeated at 3, 6 and 12 months according to the same protocol. The study was not blinded for patients or care providers, but for evaluators assessing outcomes.

Patients assessed as non-eligible were those with a very short life expectancy (shorter than 6 months), patients unable to fill in data collection material or patients that could not take part in the intervention of that study and had different impairments (eg, visual—not seeing at a distance of 3 m, hearing—not able to communicate by telephone, motor—not able to swing arm at least 10 times in a row or cognitive—assessed by a HF team).

Demographic and clinical variables of patients were gathered by self-report and review of medical records.^15^

### Measurements

Data on long-term all-cause mortality were gathered 3 years after study completion for the last included patient (June 2020) on patients included in the study in Sweden. By using the unique personal identity number assigned to each Swedish resident, date and cause of death were obtained from the Swedish Cause of Death Register held by the National Board of Health and Welfare.

Exercise capacity was measured with the 6MWT. The 6MWT was conducted according to a standardised protocol, where patients were instructed to walk at a self-selected pace in a corridor of 30 m length, marked every 3 m, for 6 min. All patients were encouraged in the same way. The American Thoracic Society stated in 2002 that the self-paced 6MWT, meaning that patients choose the intensity and are allowed to stop and rest during the test, can assess submaximal level of functional capacity. An improvement of 30 up to 50 m has previously been found in HF trials to be associated with favourable effects on morbidity and mortality.^9 10^

At first, we categorised patients into quartiles based on baseline results of the 6-Minute Walk Distance (6MWD): group 1 ≤383 m, group 2: 384–450 m, group 3: 451–527 m and group 4: ≥528 m. Due to ceiling effect, groups 3 and 4 were merged into one group, named group 3, for further analysis and results are presented according to the following groups: group 1: ≤383 m, group 2: 384–450 m and group 3: ≥451 m. In addition, based on previous evidence in terms of a clinically meaningful improvement in 6MWD, we compared participants that improved more than 30 m with those improving less than 30 m or deteriorating over a period of 12 months.^16^

HRQoL was measured using MLHFQ, which is a self-administered instrument specifically designed for the evaluation of HRQoL in patients with HF. It is composed of 21 items. Each item is scored on a Likert 6-type Scale ranging from 0 to 5 with a total score that can range from 0 to 105. MLHFQ covers two domains: a physical domain (eight items with a total score range of 0 to 40) and an emotional domain (five items with a total score range of 0 to 25). The remaining eight questions are only considered for calculating the total score. Higher scores indicate higher impairment in HRQoL. A 5-point change in score is interpreted as a clinically important difference in score.^17^

Regarding MLHFQ, patients in our study were also stratified into three groups based on tertiles of their score in the physical domain at baseline: group 1: 0–5 points, group 2: 6–12 points and group 3: 13–40 points. Thereafter, we investigated whether there was a prognostic value of the change in score over 12 months (improved: −40 to −5 points, stable: −5 to +5 points, deteriorating: +5 to +40 points).

### Statistical analyses

Patients from the intervention and the control group were analysed together in a pooled analysis.

Continuous variables are presented as mean±SD if normally distributed or median with IQR represented by the 25th and 75th percentiles if skewed distribution, and categorical variables as percentages (%). Differences in continuous variables were compared by one-way analysis of variance, when normally distributed, and by Kruskal-Wallis test if not normally distributed. Differences in categorical variables were compared by χ^2^ test. The two-tailed significance level test was set to p<0.05. Analyses of change over time were performed as complete-case analyses including only patients with available baseline and 12-month measurements. No imputation was performed due to the exploratory nature of the study. Cox regression analysis was performed to explore the impact of walked distance on all-cause mortality as per 10 m increase. Survival curves were presented using Kaplan-Meier statistics, and intergroup differences were assessed by the log-rank test.

Absolute changes for 6MWD were calculated by subtracting the baseline value from the value obtained at 12 months.

Cox proportional hazards regression was used to analyse all-cause mortality, reported as HRs, with significance defined as p<0.05 or a 95% CI excluding 1. Several models were performed to assess the association between risk factors and all-cause mortality. In step 1, univariate analyses were performed to identify potential covariates. Thereafter, the Cox regression model was adjusted for confounders (age, gender) and then further adjusted for explanatory risk factors resulting in the final multiple Cox regression model presented in table 2.

All analyses were performed with IBM 31.0 SPSS Statistics.

## Results

All the 333 HF patients in the Swedish subsample were included in the analysis. The median age was 70 (61, 76) years, and approximately 30% of the patient population were female (table 1).

**Table 1:** Demographic and clinical characteristics of patients at baseline in relation to groups of baseline 6MWT: group 1: ≤383 m, group 2: 284–450 m and group 3: ≥451 m

Characteristic	Overall	Group 1 6MWD ≤383 m	Group 2 6MWD 384–450 m	Group 3 6MWD ≥451 m	Missing data	P value
N of patients	333	82	82	163	6	
6MWT in metres	449.6 (110.4)	305.2 (71.1)	421 (20.9)	536.6 (56.3)	6	**<0.001**
Age in years	70 (61,76)	72 (67, 81)	72 (65, 76)	66 (54,72)		**<0.001**
Female gender (%)	104 (31.2%)	35 (42.7%)	30 (36.6%)	36 (22.1%)		**0.002**
BMI in kg/m^2^	27.9 (25.2, 31.9)	29.5 (24.5, 32.7)	27.7 (25.3, 32)	27.6 (25.5, 31.3)	17	0.559
Smoking (%)	15 (4.5%)	5 (6.1%)	6 (7.3%)	4 (2.5%)	5	0.173
Alcohol consumption, more than 2 glasses/week (%)	127 (38.1%)	27 (32.9%)	29 (35.3%)	68 (42.4%)	6	0.593
Ischaemic HF-aetiology (%)	127 (38.1%)	37 (45.1%)	35 (42.7%)	54 (33.1%)	12	0.122
NYHA class					13	**<0.001**
NYHA I (%)	28 (8.4%)	0	8 (9.8%)	20 (12.3%)		
NYHA II (%)	202 (60.7%)	29 (35.4%)	44 (53.7%)	123 (75.5%)		
NYHA III (%)	90 (27%)	49 (59.8%)	26 (31.7%)	15 (9.2%)		
Systolic BP (mm Hg)	120 (110,136)	120 (112, 136)	123 (110, 140)	120 (114, 130)	3	0.999
Diastolic BP (mm Hg)	72 (65,81)	71 (61, 80)	74 (65, 80)	70 (65, 85)	4	0.251
Heart rate/min	67 (60,76)	67 (63, 77)	69 (62, 77)	65 (60, 76)	3	0.071
Blood tests						
S-creatinine (umol/L)	93 (76,113)	102 (84, 118)	99 (82, 122)	87 (73, 106)	11	**<0.001**
S-sodium (mmol/L)	141 (139,142)	141 (139, 142)	140 (139, 142)	141 (139, 142)	17	0.650
S-potassium (mmol/L)	4.3 (4.1, 4.6)	4.4 (4.1, 4.7)	4.4 (4.0, 4.7)	4.2 (4.1, 4.5)	13	0.245
S-haemoglobin (g/dL)	13.7 (1.5)	13.1 (1.3)	13.6 (1.6)	13.9 (1.4)	12	**<0.001**
NT-ProBNP (pg/mL)	713 (270, 1800)	1320 (659, 2780)	1000 (340, 1885)	435 (207, 1032)	74	**<0.001**
Echocardiography						
LVEF*	35 (28,44)	35 (28,49)	40 (30,45)	35 (30,43)	124	0.721
ECG						
Sinus rhythm (%)	190 (57.1%)	39 (47.6%)	44 (53.7%)	104 (63.8%)	6	
Atrial fibrillation (%)	85 (25.5%)	30 (36.6%)	20 (24.4%)	33 (20.2%)		
PM rhythm (%)	38 (11.4%)	7 (8.5%)	10 (12.2%)	21 (12.9%)		
PM (%)	58 (17.4%)	14 (17.1%)	18 (22%)	26 (16%)	6	0.509
ICD (%)	64 (19.2%)	12 (14.6%)	21 (25.6%)	30 (18.4%)	8	0.238
CRT (%)	35 (10.5%)	8 (9.8%)	9 (11%)	18 (11%)	9	0.966
Medication						
ACEi/ARB (%)	317 (95.2%)	75 (91.5%)	78 (95.1%)	158 (96.9%)	1	0.310
Beta-blocker (%)	312 (93.7%)	77 (93.9%)	74 (90.2%)	155 (95.1%)	2	0.204
MRA (%)	164 (49.2%)	45 (54.9%)	43 (52.4%)	73 (44.8%)	2	0.405
Daily use of diuretics (%)	185 (55.6%)	60 (73.2%)	46 (56.1%)	75 (46%)	1	**<0.001**
Digoxin (%)	26 (7.8%)	6 (7.3%)	8 (9.8%)	12 (7.4%)	1	0.789
Anticoagulants (%)	151 (45.3%)	46 (56.1%)	35 (42.7%)	68 (41.7%)	1	0.065
Comorbidity						
Cerebrovascular disease (%)	32 (9.6%)	9 (11%)	8 (9.8%)	15 (9.2%)	10	0.924
Chronic pulmonary disease (%)	24 (7.2%)	13 (15.9%)	7 (8.5%)	4 (2.5%)	9	**<0.001**
Diabetes (%)	71 (21.3%)	25 (30.5%)	18 (22%)	27 (16.6%)	8	**0.047**
Diabetes with end-organ damage (%)	14 (4.2%)	8 (9.8%)	2 (2.4%)	4 (2.5%)	10	**0.017**
Moderate or severe renal dysfunction (%)	18 (5.4%)	5 (6.1%)	6 (7.3%)	7 (4.3%)	10	0.633
Cancer (%)	42 (12.6%)	16 (19.5%)	10 (12.2%)	16 (9.8%)	10	0.108
Questionnaire						
MLHFQ score, total	27 (13,46)	38 (22,57)	28 (16, 46)	21 (10,41)	8	**<0.001**
MLHFQ score, physical	12 (5,20)	17 (12, 26)	13 (6, 20)	10 (4,19)	8	**<0.001**
Alive (%), follow-up, days	285 (85.6%)	61 (74.4%)	69 (84.1%)	149 (91.4%)		**0.002**
Total amount of days in the study	1900 (1566,2271)	1924 (1557, 2253)	1933 (1633, 2244)	1897 (1565, 2298)		0.564

Continuous variables are presented as mean±SD if normally distributed or median with IQR represented by the 25th and 75th percentiles if skewed distribution, and categorical variables as percentages (%). Groups 2 and 3 were compared with group 1. Bold indicated statistical significance at p<0.05.

* Exact data on LVEF were missing in approximately 30% of patients.

ACEi/ARB, ACE inhibitor/angiotensin receptor blocker; BMI, body mass index; BP, blood pressure; CRT, cardiac resynchronisation therapy; HF, heart failure; ICD, implantable cardioverter-defribrillator; LVEF, left ventricular ejection fraction; MLHFQ, Minnesota Living with Heart Failure Questionnaire; MRA, mineralocorticoid receptor antagonist; 6MWD, 6-Minute Walk Distance; 6MWT, 6-Minute Walk Test; NT-ProBNP, N-terminal pro-B-type natriuretic peptide; NYHA, New York Heart Association Functional Class; PM, pacemaker.

The mean 6MWD at baseline was 450 (110) m. 91% of the patients (n=303) had a baseline 6MWD of >300 m and approximately 8% (n=28) had a baseline 6MWD of above 600 m. None of the patients had a 6MWD at baseline shorter than 100 m.

A total of 14.4% (n=48) of patients died during follow-up. The highest mortality (26%, n=21) was seen in Group 1 of 6MWD, followed by 16% (n=13) mortality in group 2 and group 3 (9%, n=14). Patients in group 1 according to baseline 6MWD tertiles were older, more often men, in NYHA-class III, with higher N-terminal pro-B-type natriuretic peptide (NT-ProBNP) values and more comorbidities such as diabetes and chronic pulmonary disease, than in groups 2 and 3. They also reported higher scores in MLHFQ, showcasing greater impairment in HRQoL. To further illustrate the relationship between functional capacity and both HRQoL and biomarker levels, scatter plots of baseline 6MWD versus MLHFQ score and baseline 6MWD versus NT-proBNP are presented in online supplemental file 1 and 2.

The mean change of the 6MWD between baseline and 12 months in the entire group was an increase of 6.8±65.8 m (missing values in 87 patients). In total, 16.5% of the patients (n=55) improved more than 30 m at 12 months, 46.2% (n=154) showed no clinically significant change (−30 m to +30 m), and 11.1% (n=37) deteriorated in distance walked on the 6MWT by more than 30 m.

HR was estimated at 0.96 (95% CI 0.94 to 0.98), meaning that every 10 m increase in 6MWD was associated with a 4% lower hazard of mortality.

**Figure 1:**
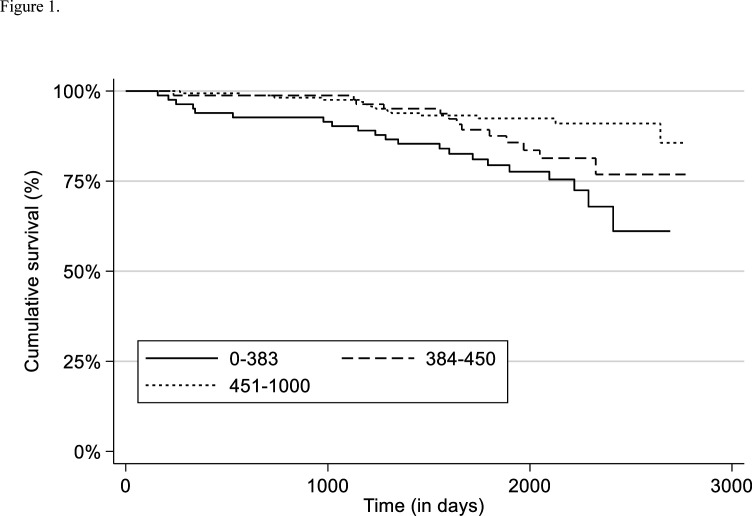
Kaplan-Meier survival plot of patients in relation to tertile groups of the baseline 6MWT (log-rank test, p=0.002). 6MWT, 6-Minute Walk Test.

Kaplan-Meier survival analysis in relation to groups of the baseline 6MWD showed significant differences between the three groups (p=0.002), as shown in figure 1.

**Figure 2:**
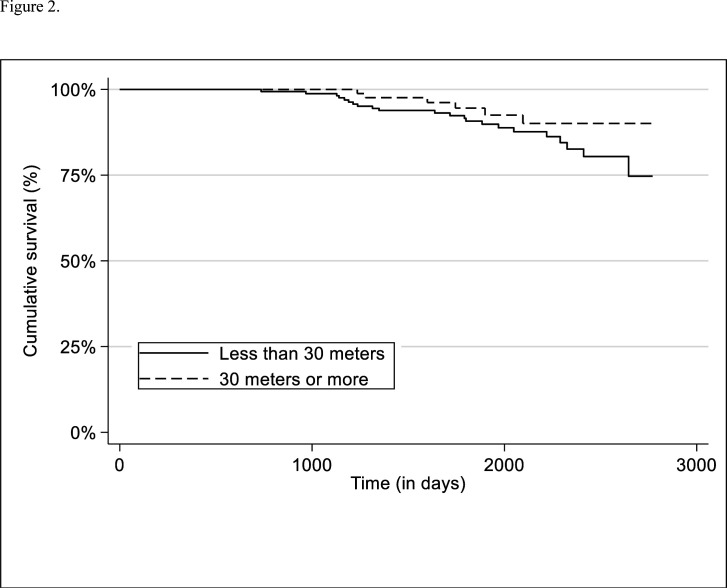
Kaplan-Meier survival plot of patients in relation to change of more than 30 m over time of the 6MWT (log-rank test, p=0.21). 6MWT, 6-Minute Walk Test.

**Figure 3:**
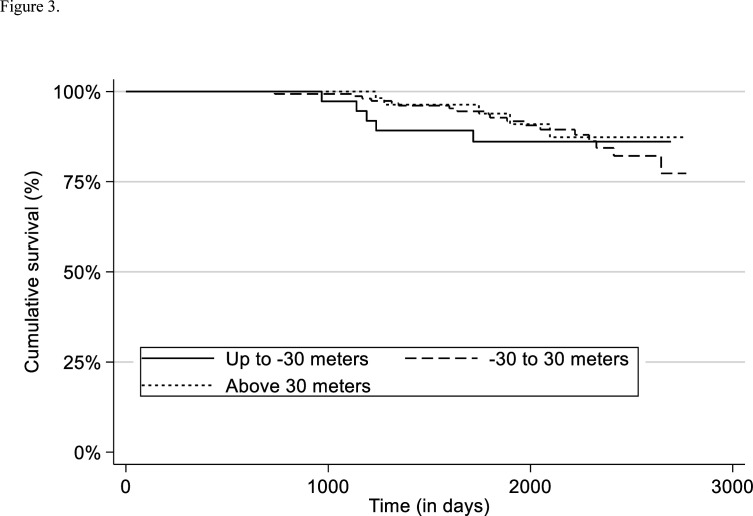
Kaplan-Meier survival plot of patients in relation to change over time of the 6MWT (log-rank test, p=0.82). 6MWT, 6-Minute Walk Test.

There was no significant difference in survival between patients that improved more than 30 m (n=55) compared with those improving less than 30 m or deteriorating over a period of 12 months (n=191) (p=0.21), as shown in figure 2, and neither on improvement, stability or deterioration over a 12-month period (p=0.82), as presented in figure 3.

Six variables were significantly associated with all-cause mortality in univariate analysis with p<0.05 (table 2). In the Cox proportional hazards regression analyses, age was significantly associated with adverse outcomes (HR 1.06, 95% CI 1.03 to 1.09, p<0.001), corresponding to a 6% higher hazard per additional year of age. Body mass index (BMI) showed a borderline protective effect, with higher BMI being associated with lower hazard (HR 0.94, 95% CI 0.88 to 1.00, p=0.051). NT-proBNP was a strong predictor of adverse outcome (χ²=13.90, p<0.001). A diagnosis of diabetes mellitus was associated with a significantly higher hazard of mortality (HR 2.40, 95% CI 1.33 to 4.34, p=0.004).

**Table 2:** Univariate and multivariate Cox regression analysis of time to death

	Univariate regression				Multivariate regression			
	HR	95% CI for HR		P value	HR	95% CI for HR		P value
		Lower	Upper			Lower	Upper	
Demographics								
Gender (female vs male)	0.58	0.30	1.15	0.12	0.55	0.23	1.34	0.19
Age (per year)	1.06	1.03	1.09	**<0.001**	1.04	1.00	1.08	**0.02**
BMI (per unit)	0.94	0.88	1.00	0.05				
Marital status (divorced/single vs married/sambo)	0.78	0.39	1.53	0.46				
Smoking (yes vs no)	0.75	0.23	2.41	0.63				
Clinical characteristics								
Ischaemic aetiology of HF	0.64	0.36	1.14	0.13				
NYHA class II vs I	4.24	0.58	31.16	0.16	2.72	0.36	20.62	0.33
NYHA III vs I	3.75	0.49	28.83	0.21	1.31	0.16	10.64	0.79
Systolic BP	1.00	0.99	1.02	0.77				
AF vs SR	1.24	0.62	2.49	0.54				
Laboratory values								
S-sodium	1.02	0.91	1.14	0.71				
S-creatinine	1.01	1.00	1.02	**0.004**				
S-haemoglobin	0.80	0.66	0.97	**0.02**				
NT-ProBNP (Groups)	3.68	1.79	7.58	**<0.001**	2.80	1.29	6.09	**0.009**
Treatment								
ICD (yes vs no)	1.00	0.48	2.09	0.99				
CRT	0.63	0.28	1.42	0.27				
ACEi/ARB	1.00	0.24	4.14	0.99				
Beta-blockers	1.67	0.60	4.66	0.33				
MRA	0.80	0.45	1.42	0.44	2.07	1.05	4.09	**0.04**
Comorbidities								
COPD	1.16	0.36	3.73	0.81				
DM (yes vs no)	2.40	1.33	4.34	**0.004**	2.51	1.29	4.85	**0.006**
Moderate or severe renal disease	0.45	0.16	1.26	0.13				
Cancer, any type	1.13	0.45	2.87	0.79				

Bold indicated statistical significance at p<0.05.

NT-ProBNP groups: cut-off 713 (median value).

ACEi/ARB, ACE inhibitor/angiotensin receptor blocker; AF, atrial fibrillation; BMI, body mass index; BP, blood pressure; COPD, chronic obstructive pulmonary disease; CRT, cardiac resynchronisation therapy; DM, diabetes mellitus; HF, heart failure; ICD, implantable cardioverter-defribrillator; MRA, mineralocorticoid receptor antagonist; NT-ProBNP, N-terminal pro-B-type natriuretic peptide; NYHA, New York Heart Association Functional Class; PM, pacemaker; SR, sinus rhythm.

In multivariate Cox proportional regression model age, NT-ProBNP, treatment with MRA and suffering from diabetes mellitus remained significant independent predictors of mortality. Specifically, NT-ProBNP was moderately correlated with distance walked at 6MWT (Pearson correlation r=−0.346, p<0.001), but results need to be interpreted with caution due to approximately 22% missing values.

The mean change of the physical domain of MLHFQ between baseline and 12 months in the entire group was an improvement −0.43±7.8 (missing values in 73 patients). In total, 19.5% of the patients (n=65) improved at 12 months, 45.9% (n=153) were stable and 12.6% (n=42) deteriorated.

**Figure 4:**
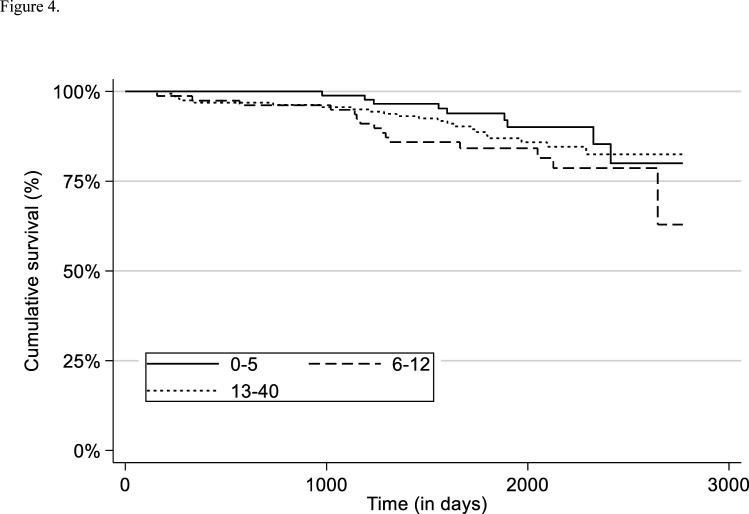
Kaplan-Meier survival plot of patients in relation to baseline score in the physical domain of baseline MLHFQ (log-rank test, p=0.187). MLHFQ, Minnesota Living with Heart Failure Questionnaire.

**Figure 5:**
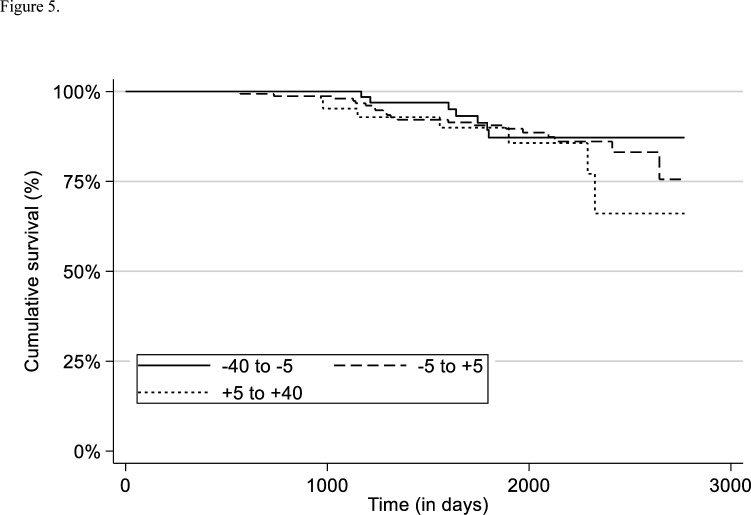
Kaplan-Meier survival plot of patients in relation to change over time in the physical domain of MLHFQ (log-rank test, p=0.554). MLHFQ, Minnesota Living with Heart Failure Questionnaire.

Kaplan-Meier survival analysis in relation to tertile groups of the baseline score on physical domain of MLHFQ did not show significant differences between the three groups (p=0.187), as shown in figure 4 as well as in relation to change over time of the same score (p=0.554), as presented in figure 5.

## Discussion

The main finding of this prospective study of clinically stable patients with chronic HF is that the baseline 6MWD provides significant prognostic information for long-term mortality. In contrast, the baseline value of the physical scale of MLHFQ along with changes over time in either 6MWD or HRQoL was not associated with survival. Among the clinical and biochemical variables investigated, NT-ProBNP emerged as the only independent predictor of adverse outcome in multivariate analysis.

In everyday practice, there is a need for prognostic tools that can easily be part of patients’ evaluation. The implementation of prognostic tools will be easier and more successful with the utilisation of measurements and evaluations already used in clinical practice and easily performed and reproduced. Regarding 6MWT the evidence has been inconsistent, as older studies could not prove an association to survival outcomes^10^ while more recent studies have shown prognostic usefulness.^18 19^ According to several studies evaluating 6MWT in HF patients, a threshold of 300 or less walked metres in 6MWT is indicative of poor physical performance and a worse prognosis. A recent meta-analysis with 22 598 patients with HF has shown that patients with HF with poor physical functional performance at 6MWT had an increased risk of all-cause mortality and an increased risk of HF mortality.^20^

The findings of our study confirm prior evidence on the prognostic value of exercise capacity in HF. In our study, patients in the lowest 6MWD group have higher mortality compared with the remaining groups, supporting the concept that reduced functional capacity reflects the overall disease severity. Patients included in this study is a relatively high-functioning HF population. The lack of association between 6MWD change and mortality may be explained by the relatively small mean change in walking distance over 1 year and the limited number of events, but may also indicate that it is the baseline physical capacity—and not its change over time—that carries the strongest prognostic information.

In a recent Norwegian HF registry including over 5000 clinically stable outpatients with HF, Grundtvig *et al* demonstrated that baseline 6MWD was a strong and independent predictor of all-cause mortality, even after adjustment for NT-proBNP and conventional clinical variables.^8^ In line with these findings, our study confirms the prognostic importance of baseline 6MWD, showcasing that patients in the lowest group of 6MWD had substantially higher mortality in comparison to those in the upper groups. However, unlike the Norwegian cohort, we did not observe an association between longitudinal changes in 6MWD and survival. Similar findings to the Norwegian study have been recently presented by Atias *et al*, suggesting that repeated functional capacity testing may refine mortality prediction in HF patients.^21^ This could possibly reflect differences in study design, patient characteristics, as well as the smaller number of events in our population. It could also suggest that baseline physical capacity provides the most robust prognostic information in clinically stable HF patients.

Although neither the baseline score of the physical dimension of MLHFQ nor its change over time reached statistical significance, we observed a trend suggesting that improvements in patient-reported physical function may be associated with better outcomes. Although MLHFQ was not associated with mortality in this study, this likely reflects that patient-reported QoL captures symptom burden and functional limitations that are only partly aligned with the biological determinants of survival. In a relatively stable and well-treated population, its prognostic value may be limited. Nevertheless, MLHFQ remains clinically important as a complementary measure, providing unique insight into the patient experience and supporting a more comprehensive, patient-centred assessment.

Future studies with larger cohorts are warranted. Another aspect to consider is that many studies to date use the Kansas City Cardiomyopathy Questionnaire to measure disease-specific QoL in HF.^22^ This instrument has shown a better predictive value and higher responsiveness to change than MLHFQ.^23^

In multivariate Cox regression, NT-proBNP, age and diabetes mellitus were independent predictors of all-cause mortality. Patients with NT-proBNP values above the median had almost a threefold higher risk of death. This aligns with evidence establishing NT-proBNP as one of the most powerful prognostic biomarkers in HF. The borderline association of gender and BMI with outcome is consistent with previously reported trends suggesting better survival in women and in patients with higher BMI, although these effects did not reach statistical significance in our study, possibly due to the limited sample size and small number of events.

### Limitations

First, our study is a secondary analysis of the international HF-Wii study, analysing data only from a Sweden-specific population, that may impact the generalisability of the study results towards other countries. However, studies on HF patients carried out in other Nordic countries seem to have consistent results.^8^ Second, this study, although it included patients over the whole range of ejection fraction, has a moderate sample size and number of events, which may have limited the statistical power to detect associations for categorical variables. Patients with shortened life expectancy may be underrepresented. Third, the analysis was based on data gathered from the HF-Wii study, where patients were well treated with a high percentage of patients receiving ACEi/angiotensin receptor blocker, beta-blockers and MRA, according to current guidelines then. At the time of inclusion, treatment with SGLT2-inhibitors was not standard of care. Moreover, patients had a rather high baseline distance on the 6MWT reflecting a higher level of functional capacity. Lastly, although the 6MWT has been found to be reproducible, intrinsic unreliability may be a limitation. The 6MWT protocol specifies the allowed motivation extent. However, individual factors as well as factors that may be affected during the interaction between the patient and the tester may influence the results.

### Strengths of the study and clinical implications

Our findings underscore the value of the baseline 6MWD and NT-proBNP in prognostic assessment of ambulatory HF patients. The possibility of combined use of the 6MWT and MLHFQ presents an easily performed and effective approach for risk assessment in patients with HF, offering both objective and subjective perspectives on present patient status, while requiring minimal resources, which facilitates their clinical use. When combined with active patient engagement, it promotes patient-centred care and enhances clinical decision-making. Positive effects may be improved adherence and earlier detection of possible clinical deterioration. Repeated testing of exercise capacity and HRQoL remain valuable for clinical monitoring, but their prognostic utility appears limited compared with baseline evaluation and biomarkers.

## Conclusions

The baseline 6MWD and NT-proBNP levels are strong predictors of mortality, whereas changes over time in exercise capacity and HRQoL were not. The result emphasises their use in the prognostic evaluation of patients with HF irrespective of their ejection fraction.

## Supplementary material

10.1136/openhrt-2026-004130 online supplemental file 1

10.1136/openhrt-2026-004130 online supplemental file 2

## Data Availability

Data are available on reasonable request.
