# Supplementary figures and images for: Prognostic value of baseline and longitudinal changes in exercise capacity and quality of life in the HF-Wii Swedish population
Source: Open Heart. 2026 Jul 8;13(2):e004130. doi: 10.1136/openhrt-2026-004130 (PMC13347824; doi:10.1136/openhrt-2026-004130)

Scatter plot for the relation between MLHFQ and 6MWD


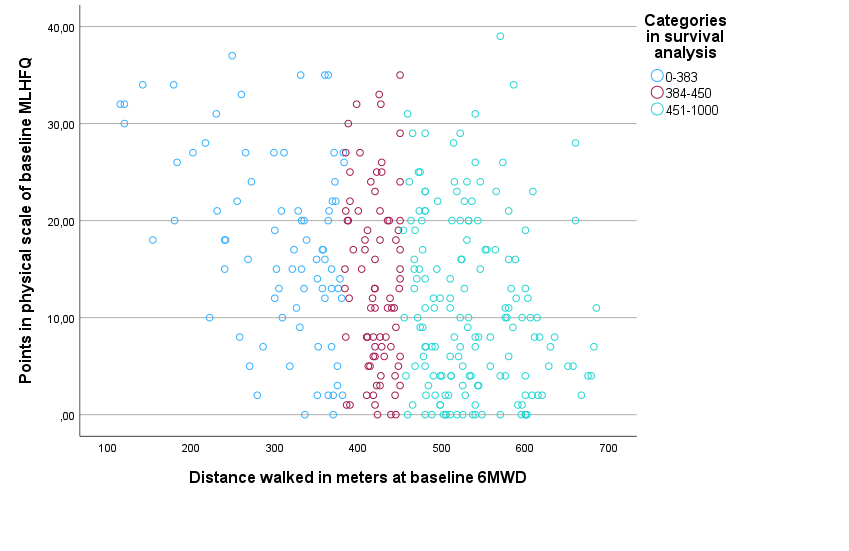

Supplement: online supplemental file 1 [file openhrt-13-2-s001.docx]

Scatter plot for the relation between NT-proBNP and 6MWD


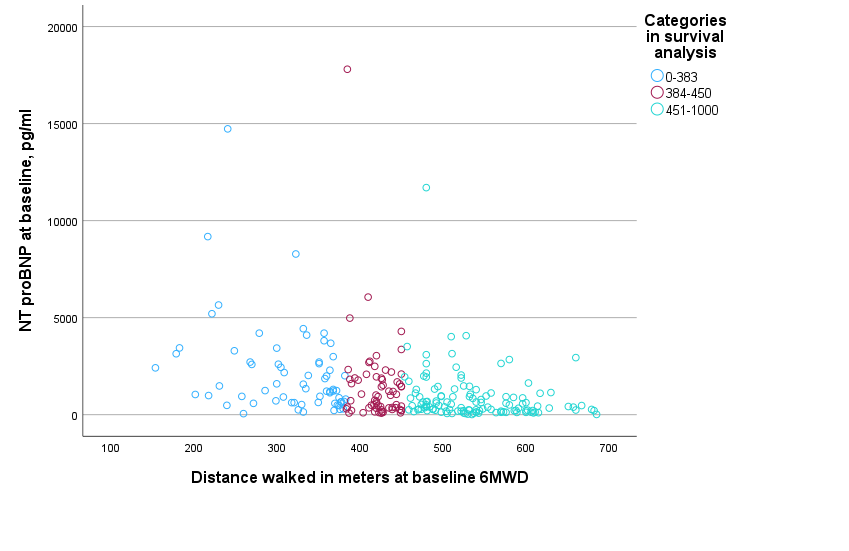

Supplement: online supplemental file 2 [file openhrt-13-2-s002.docx]
